# Supplementary material for: NR2F1, a Tumor Dormancy Marker, Is Expressed Predominantly in Cancer-Associated Fibroblasts and Is Associated with Suppressed Breast Cancer Cell Proliferation
Source: Cancers (Basel). 2022 Jun 15;14(12):2962. doi: 10.3390/cancers14122962 (PMC9220877; doi:10.3390/cancers14122962)
Supplement: Supplementary file 1 [file cancers-14-02962-s001.zip › cancers-1746495-supplementary.pdf]

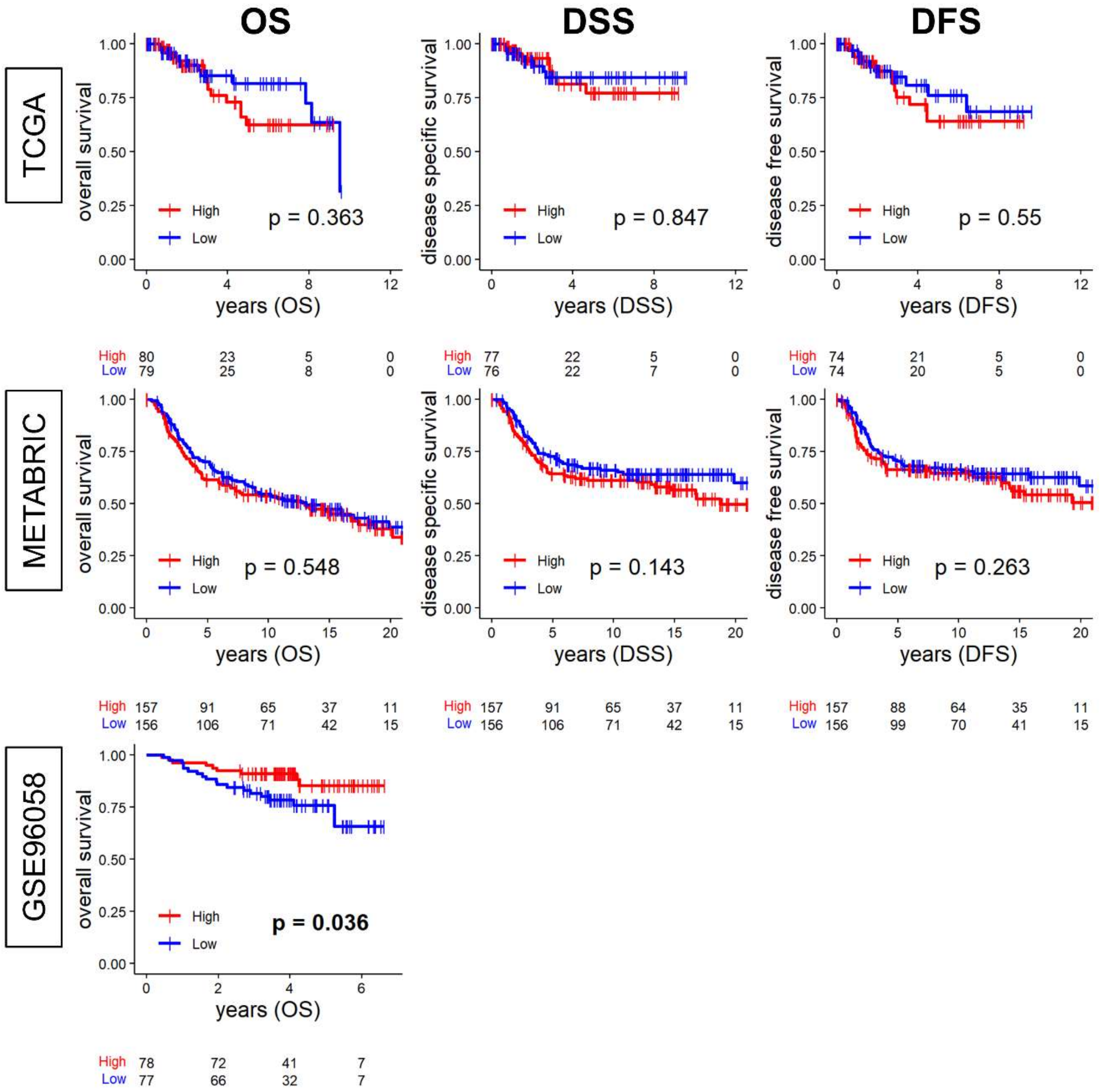

Supplementary Figure S1. Survival analysis between high and low *NR2F1* groups in triple-negative breast cancer. Kaplan–Meier curves of overall survival (OS), disease-specific survival (DSS), and disease-free survival (DFS) based on high and low *NR2F1* expression in triple-negative breast cancer of three large cohorts. Log-rank test was used for the analysis, and significant p values are shown in bold. $p < 0.05$ was considered significant.

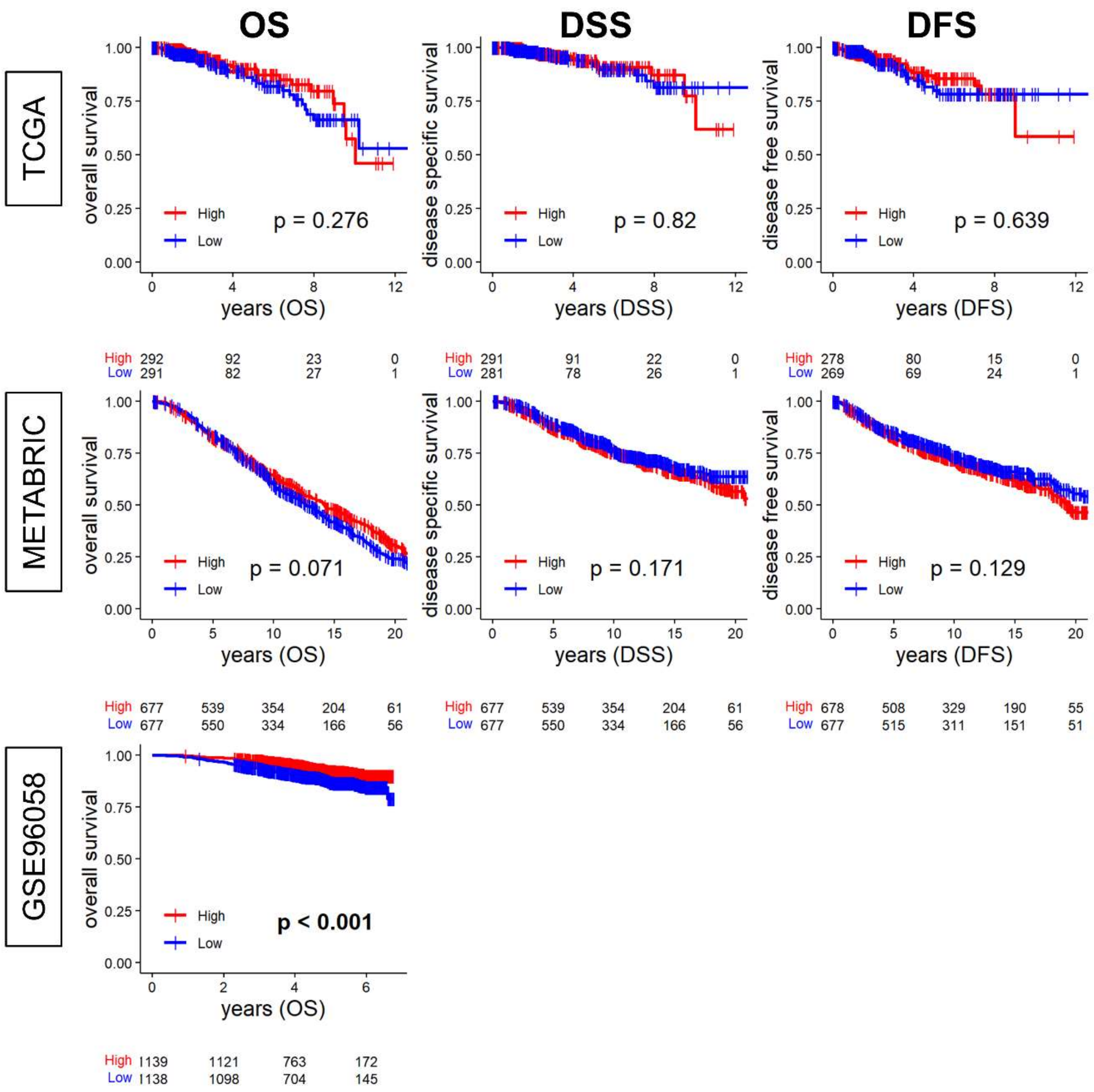

Supplementary Figure S2. Survival analysis between high and low *NR2F1* groups in ER-positive and HER2-negative breast cancer. Kaplan–Meier curves of OS, DSS, and DFS based on high and low *NR2F1* expression in ER-positive and HER2-negative breast cancer of three large cohorts. Log-rank test was used for the analysis, and significant p values are shown in bold. $p < 0.05$ was considered significant.

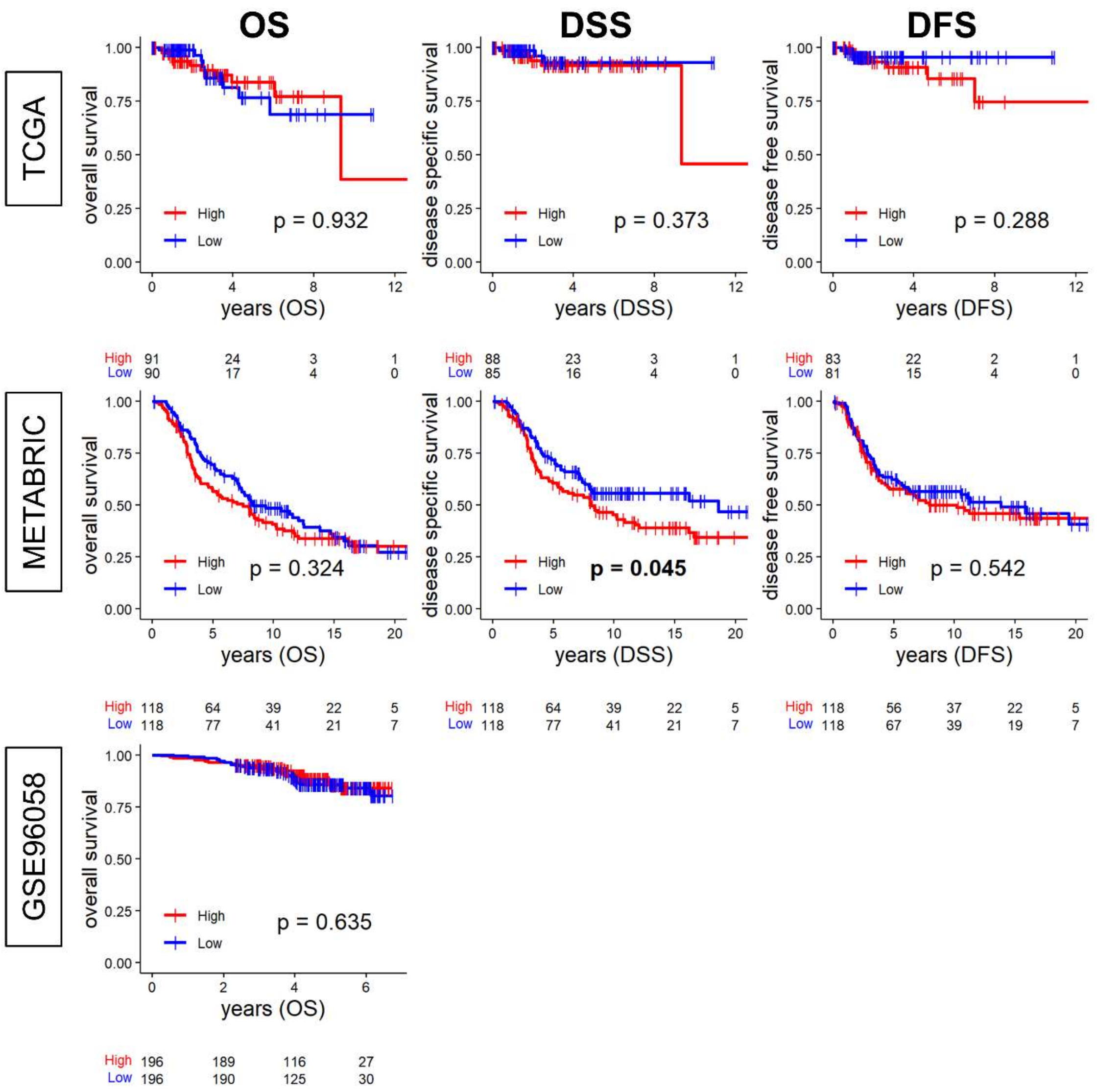

Supplementary Figure S3. Survival analysis between high and low *NR2F1* groups in HER2-positive breast cancer. Kaplan–Meier curves of OS, DSS, and DFS based on high and low *NR2F1* expression in HER2-positive breast cancer of three large cohorts. Log-rank test was used for the analysis, and significant p values are shown in bold. $p < 0.05$ was considered significant.

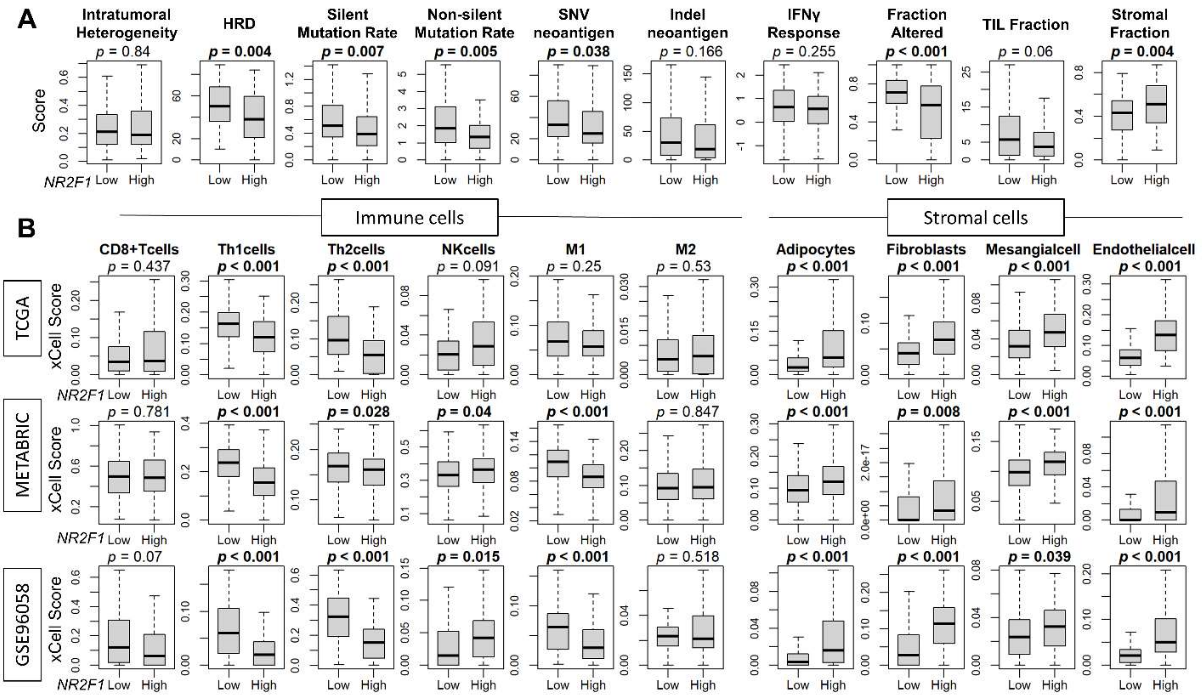

Supplementary Figure S4. Association of *NR2F1* with immunity within the tumor microenvironment of triple-negative breast cancer. (A) Boxplots showing various scores based on high and low *NR2F1* expression in triple-negative breast cancer of TCGA. Intratumoral heterogeneity, homologous recombination deficiency (HRD), silent/non-silent mutation rate, SNV/Indel neoantigen, interferon gamma response, fraction altered, tumor-infiltrating lymphocytes (TIL) fraction, and stromal fraction. **(B)** Boxplots showing immune and stromal cell fractions between *NR2F1* high and low groups in triple-negative breast cancer of three large cohorts. Mann–Whitney U test was used to compare the two groups and p values are shown in bold for significant results ($p < 0.05$).

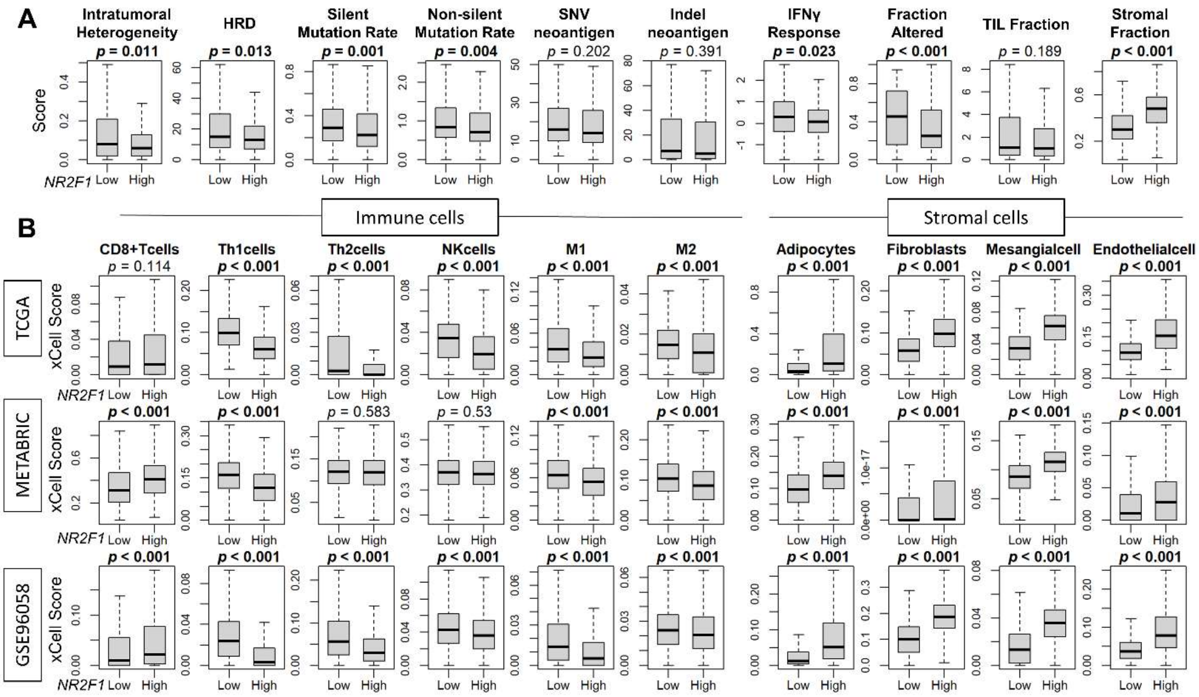

Supplementary Figure S5. Association of *NR2F1* with immunity within the tumor microenvironment of ER-positive and HER2-negative breast cancer. (A) Boxplots showing various scores based on high and low *NR2F1* expression in ER-positive and HER2-negative breast cancer of TCGA. Intratumoral heterogeneity, homologous recombination deficiency (HRD), silent/non-silent mutation rate, SNV/Indel neoantigen, interferon gamma response, fraction altered, tumor-infiltrating lymphocytes (TIL) fraction, and stromal fraction. **(B)** Boxplots showing immune and stromal cell fractions between *NR2F1* high and low groups in ER-positive and HER2-negative breast cancer of three large cohorts. Mann–Whitney U test was used to compare the two groups and p values are shown in bold for significant results ($p < 0.05$).

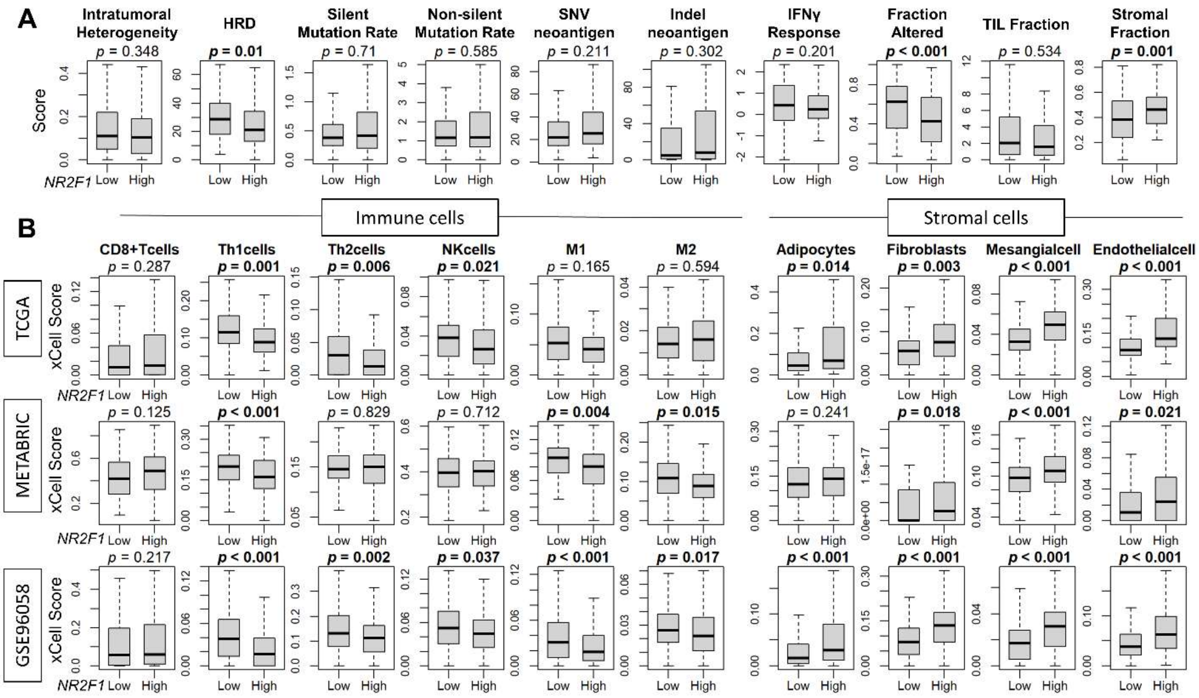

Supplementary Figure S6. Association of *NR2F1* with immunity within the tumor microenvironment of HER2-positive breast cancer. (A) Boxplots showing various scores based on high and low *NR2F1* expression in HER2-positive breast cancer of TCGA. Intratumoral heterogeneity, homologous recombination deficiency (HRD), silent/non-silent mutation rate, SNV/Indel neoantigen, interferon gamma response, fraction altered, tumor-infiltrating lymphocytes (TIL) fraction, and stromal fraction. **(B)** Boxplots showing immune and stromal cell fractions between *NR2F1* high and low groups in HER2-positive breast cancer of three large cohorts. Mann–Whitney U test was used to compare the two groups and p values are shown in bold for significant results ($p < 0.05$).

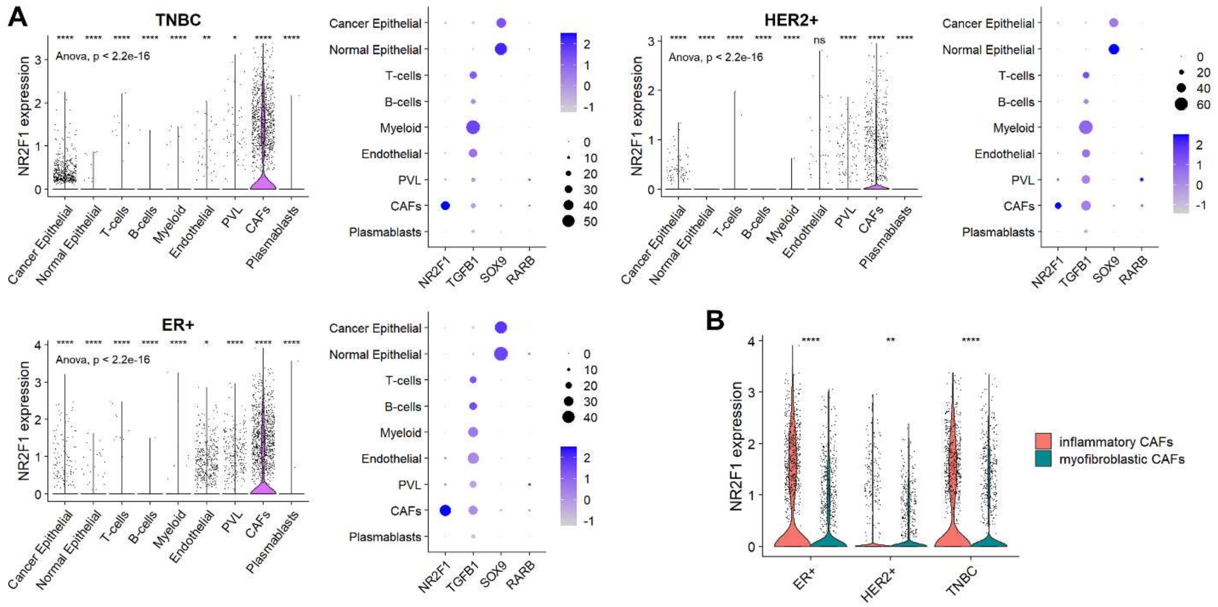

Supplementary Figure S7. Expression of *NR2F1* and dormancy-related genes by immunohistological breast cancer subtypes in Cohort 2. (A) Violin plots showing *NR2F1* expression by cell type in triple-negative (TNBC), HER2-positive (HER2+), and ER-positive HER2-negative (ER+) tumors in single-cell Cohort 2. One dot represents one cell. After multi-group comparison, baseline *NR2F1* expression and expression in each cell type were compared in two groups by the one-way ANOVA test. In addition, dot plots show the expression of *NR2F1*, *TGFB1*, *SOX9*, and *RARB* by cell type in each immunohistological subtype in single-cell Cohort 2. The size of each dot indicates the number of cells, and the purple intensity indicates the expression level. **(B)** Violin plot shows *NR2F1* expression in inflammatory CAFs (iCAFs) and myofibroblasts (myCAFs) by immunohistological subtypes in single-cell Cohort 2. Symbols in the figure are as follows: ns, $p > 0.05$; *, $p \leqq 0.05$; **, $p \leqq 0.01$; ***, $p \leqq 0.001$; ****, $p \leqq 0.0001$.

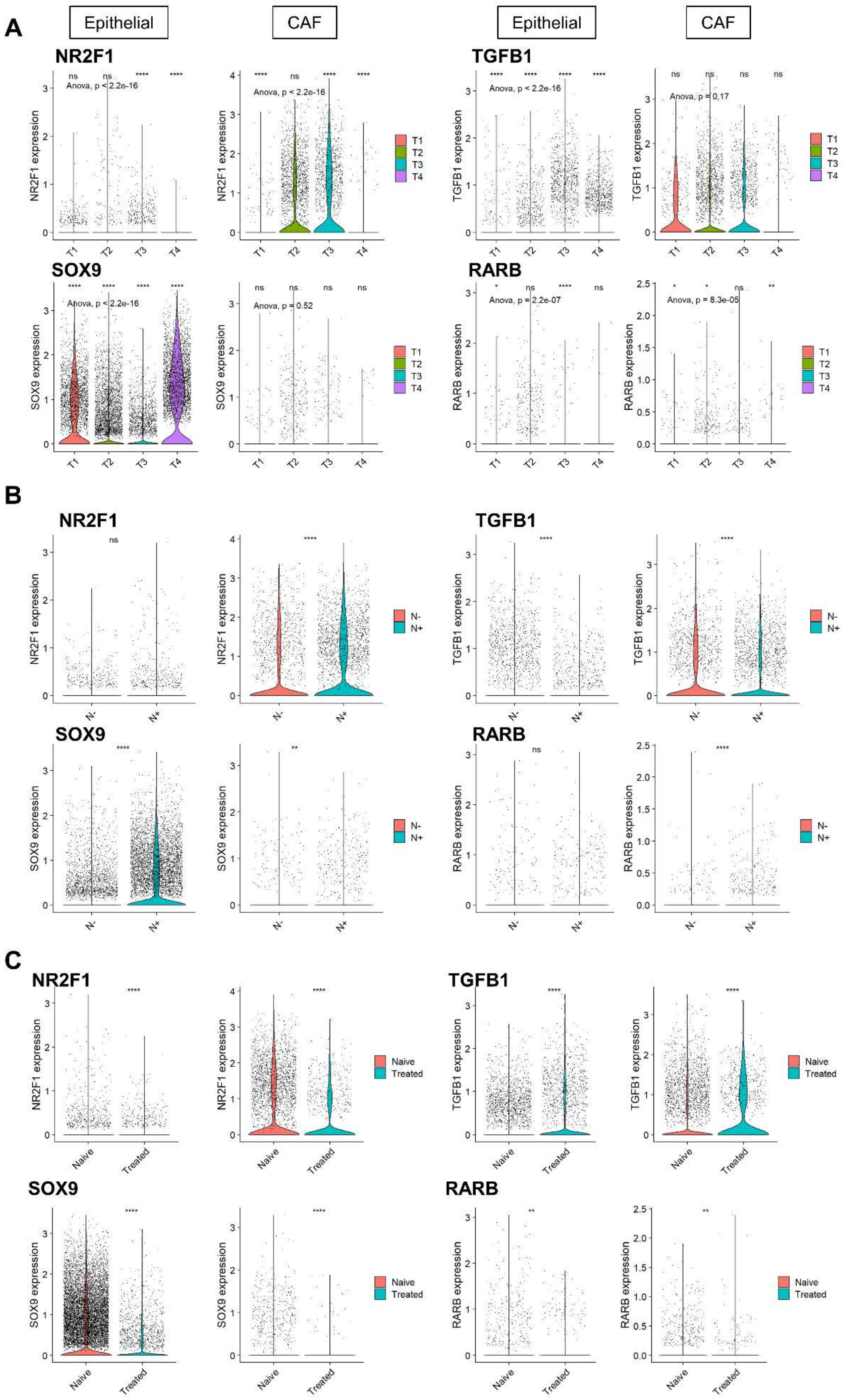
A
Epithelial
CAF
Epithelial
CAF
NR2F1
Anova, p < 2.2e-16
Anova, p < 2.2e-16
NR2F1 expression
T1
T2
T3
T4
TGFB1
Anova, p < 2.2e-16
Anova, p = 0.17
TGFB1 expression
SOX9
Anova, p < 2.2e-16
Anova, p = 0.52
SOX9 expression
RARB
Anova, p = 2.2e-07
Anova, p = 8.3e-05
RARB expression
B
NR2F1
TGFB1
SOX9
RARB
N-
N+
C
NR2F1
TGFB1
SOX9
RARB
Naive
Treated

Supplementary Figure S8. Expression of *NR2F1*, *TGFB1*, *SOX9*, and *RARB* in cancer cells and CAFs in Cohort 2. Violin plots showing *NR2F1*, *TGFB1*, *SOX9*, and *RARB* expression in epithelial cells and CAF in the single-cell Cohort 2 by **(A)** tumor size of the AJCC TNM staging, **(B)** with and without lymph node metastasis, and **(C)** with and without prior drug therapy. One dot represents one cell. After multi-group comparison, baseline *NR2F1* expression and expression in each cell type were compared in two groups by the one-way ANOVA test. Symbols in the figure are as follows: ns, p > 0.05; *, p ≦ 0.05; **, p ≦ 0.01; ***, p ≦ 0.001; ****, p ≦ 0.0001.

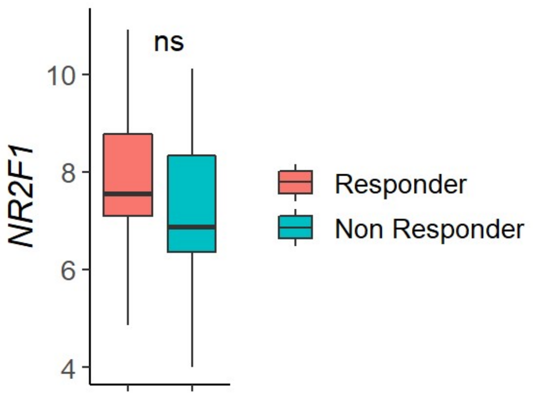

Supplementary Figure S9. Expression of *NR2F1* between endocrine therapy responder and non-responder in hormone-positive primary breast cancer. Boxplots showing *NR2F1* expression in responder and non-responder groups to the neoadjuvant endocrine therapy in GSE145325. Mann–Whitney U test was used for analysis. Symbol in the figure is as follows: ns, p > 0.05.

Supplementary Table S1: Details of neoadjuvant chemotherapy or endocrine therapy used in each cohort

*A, doxorubicin; C, cyclophosphamide; E, epirubicin; F, fluorouracil; NAC, neoadjuvant chemotherapy; nP , nab-paclitaxel; T, docetaxel.

Accession No.	Annotation No.	Type of tissue	Year	Treatment details	Numbers of patients		Numbers of patients	
					Before	After	Responder	Non-responder
GSE180280	GPL18573	Biopsy before and surgical specimen after NAC	2021	FEC +/- taxane and anti-HER2 therapy	30	7		
GSE87455	GPL10558	Paired biopsy before and surgical specimen after NAC	2017	ED (± bevacizumab)	122	70		
GSE28844	GPL570	Biopsy before NAC and surgical specimen after NAC	2012	Anthracycline and taxane (± trastuzumab)	32	29		
GSE21974	GPL6480	Paired biopsy before and after NAC	2010	EC + T	32	25		
GSE145325	GPL21290	Paired sample before neoadjuvant endocrine therapy	2020	Letrozole			35	22
